# Environmental and Behavioural Determinants of Leptospirosis Transmission: A Systematic Review

**DOI:** 10.1371/journal.pntd.0003843

**Published:** 2015-09-17

**Authors:** Mwanajaa Abdalla Mwachui, Lisa Crump, Rudy Hartskeerl, Jakob Zinsstag, Jan Hattendorf

**Affiliations:** 1 Department of Epidemiology and Public Health, Swiss Tropical and Public Health Institute, Basel, Switzerland; 2 University of Basel, Basel, Switzerland; 3 Royal Tropical Institute (KIT), KIT Biomedical Research, Amsterdam, The Netherlands; University of Tennessee, UNITED STATES

## Abstract

**Background:**

Leptospirosis is one of the most widespread zoonotic diseases, which is of global medical and veterinary importance, and also a re-emerging infectious disease. The main tracks of transmission are known; however, the relative importance of each of the components and the respective environmental risk factors are unclear. We aimed to assess and specify quantitative evidence of environmental risks of leptospirosis transmission.

**Methods/findings:**

A database of pre-selected studies, with publication dates from 1970 until 2008, was provided by an expert group. The database has been updated until 2015 using a text mining algorithm. Study selection was based on stringent quality criteria. A descriptive data analysis was performed to calculate the medians of the log transformed odds ratios. From a selection of 2723 unique publications containing information on leptospirosis, 428 papers dealing with risk factors were identified. Of these, 53 fulfilled the quality criteria, allowing us to identify trends in different geo-climatic regions. Water associated exposures were, with few exceptions, associated with an increased leptospirosis risk. In resource poor countries, floods and rainfall were of particular importance, whereas recreational water activities were more relevant in developed countries. Rodents were associated with increased leptospirosis risk, but the variation among studies was high, which might be partly explained by differences in exposure definition. Livestock contact was commonly associated with increased risk; however, several studies found no association. The median odds ratios associated with dog and cat contacts were close to unity. Sanitation and behavioural risk factors were almost always strongly associated with leptospirosis, although their impact was rarely investigated in Europe or North America.

**Conclusion:**

This review confirms the complex environmental transmission pathways of leptospirosis, as previously established. Although, floods appeared to be among the most important drivers on islands and in Asia, the consistent pattern observed for exposure to rodents and behavioural and sanitation related risk factors indicate potential areas for intervention.

## Author Summary

Leptospirosis is a bacterial disease passed from animals to people—either through direct contact with animals or indirectly via the environment. The disease can be found worldwide but is more important in tropical and subtropical countries. Due to their sheer genetic diversity, virtually all mammals can be infected by leptospires. People can be infected from a broad range of animals including livestock, pets and rodents. Transmission pathways differ among animal species as well as across rural and urban landscapes and environmental conditions which influence survival of pathogenic leptospira in surface water and moist soil. Consequently, spatial and temporal patterns of disease incidence are complex; however, a better understanding is crucial for the planning and implementation of effective interventions. We systematically reviewed the scientific literature to identify regional risk factors and assess their importance. We found considerable heterogeneity among studies indicating that epidemiological patterns are highly setting specific. Floods and heavy rain appeared to be among the most important drivers on islands and in Asia. Exposure to rodents as well as behaviour and sanitation are often important risk factors indicating potential areas for interventions.

## Introduction

Leptospirosis, classified as a neglected tropical disease, is caused by a pathogenic spirochete bacterium of the genus *Leptospira* [1],[2]. Most mammalian species can carry leptospirosis, with no disease or only mild clinical manifestations in the maintenance hosts because they are highly adapted [3]. Infection in humans is acquired through direct or indirect exposure to the urine of carrier animals. The three main tracks presenting transmission risk can be summarized as water-based, rodent-borne and livestock/pet-borne infection [1]. Humans are considered to be a dead-end host and are highly susceptible to infection with many serovars [2]. Climatic conditions strongly influence the transmission of leptospires, which require warm, humid conditions for survival. The bacteria persist for weeks to months following their excretion into water or moist soil [1]. Geologic and geographic characteristics, in conjunction with demographic, agricultural and livestock system factors, also determine transmission [4]. Rats are thought to be the main reservoir transmitting the disease to humans. However, host-pathogen relationships may change depending on contextual conditions, complicating the stipulation of a “main reservoir”. The cycles involving rodents, wildlife, livestock and pet animals vary within contaminated environments.

While humans can be infected from any of these sources with varying magnitude, they play a subordinate role in the transmission cycle. Livestock are an important source of infection for farmers, veterinarians and workers handling meat, while wildlife and rodents are the primary sources for hunters, pastoralists and urban dwellers [4].While leptospirosis is endemic among subsistence farmers who cultivate rice or engage in small scale livestock production, recreational exposure to contaminated water has become more important for sport enthusiasts, swimmers and travellers from industrialized countries. Exposure to wastewater and garbage is a risk for inhabitants of slums, reflecting the socio-economic dimension in urban and peri-urban settings of developing countries [3],[5],[6].

Not all of the described conditions are present in every geographic setting. Barriers to determining the actual burden of leptospirosis include scarcity of appropriate diagnostic testing, lack of veterinary surveillance systems to detect animal cases perhaps, lack of education among the general population as well as medical professionals and absence of environmental surveillance for identification of circulating serovars [7],[8]. Current trends of leptospirosis outbreaks, especially in endemic areas, indicate that geographic spread and epidemics will increase in the future [8]. There is a misperception that the burden and impact of leptospirosis on society are low [9]. A possible reason for this is the non-specific signs and symptoms, which mimic other infectious diseases making misdiagnosis of leptospirosis highly likely.

This study was commissioned by the Leptospirosis Burden Epidemiology Reference Group (LERG) of the World Health Organization (WHO) to summarise the pre-existing evidence on environmental risks for leptospirosis to identify the major determinants of transmission. A better understanding of context specific main transmission pathways would provide background information to assess the potential of cross-sector interventions, to develop a leptospirosis transmission model and to identify important knowledge gaps.

## Methods

A database of pre-selected studies, published from 1.1.1970 to 31.8.2008, was provided by the Leptospirosis Burden Epidemiology Reference Group (LERG), an expert advisory group to the World Health Organization (WHO). Thirty-two electronic databases were combined, for a total of 12,025 reports or publications. Details on the search algorithm are provided elsewhere[10]. Of these, 2723 articles contained information on human leptospirosis and were used as the data source for the systematic review [9]. The database has been updated for articles published between 1.9.2008 and 31.3.2015 using a Markov Chain text mining algorithm. Details on the update process are provided in S2 Text. The PRISMA checklist is provided in S1 checklist.

### Eligibility criteria

Studies which adequately described the case selection procedure and met the leptospirosis case definition were included. Leptospirosis cases were defined as including a description of clinical signs or symptoms in addition to laboratory confirmation. Clinical signs and symptoms included fever, myalgia, headache, jaundice/hepatic abnormality, renal syndrome, uveitis/ocular signs, bleeding disorders or neurologic signs. Laboratory confirmation included culture of *Leptospira* spp., microscopic agglutination test (MAT), polymerase chain reaction (PCR) or enzyme-linked immunosorbent assay (ELISA). Cross sectional, case-control and cohort studies were considered eligible, whereas case reports, reviews and outbreak reports and those not about risk factors for leptospirosis were excluded. Finally, sufficient data had to be provided so that odds ratios could be extracted or calculated.

### Study selection

Studies were classified as irrelevant, relevant or uncertain by two independent reviewers on the basis of title and abstract. Studies where risk factors were mentioned but not clearly described in the abstract were classified as uncertain and included for further clarification through full text review.

### Data extraction process

Data extracted from the eligible studies included identifying variables (database identification number, first author name, year published, title, study country), design and method variables (study type, time perspective, study population characteristics, number of subjects, method of statistical analysis) and outcomes related to risk factor variables (odds ratios, confidence limits, number of infected and non-infected, exposed and non-exposed). The study type was classified as case-control, cross-sectional, cohort or other, and time perspective was recorded as prospective or retrospective. Study population characteristics were noted, including sampling method (simple random, cluster sampling, cluster sampling proportional to size, non-random or not described), study population (field survey or hospital based study) and population type (rural, urban).

Risk factors were classified into the following categories: i) water related: flooded areas, walking through puddles, standing water present near dwellings, recent rainfall, high annual rainfall, swallowing water, swimming or fishing in stagnant water and recreational activities such as triathlon, kayaking and canoeing; ii) agriculture related: rice production, other crops and subsistence cropping in home gardens; iii) landscape factors: forest cover and rural versus urban zones; iv) socio-economic status: assessed through use of specific home construction materials as a proxy; v) sanitation: type of and proximity to sewage systems or open defecation, presence of garbage, household sanitation (presence of latrine, indoor water supply) and indoor occupation; vi) behavioural: walking barefoot, forest work or gathering fire wood, presence of wounds and the use of protective clothing; vii) animals: small mammals, livestock, cows, pigs, poultry, pets and wildlife (specifically exposure through hunting)

### Synthesis of results

Study quality was assessed by considering the case definition and method of statistical analysis. Each study was assigned a case definition quality based on the case confirmation criteria. Studies which described clinical signs and reported confirmatory microbial culture were labelled quality 1. Studies which described clinical signs and paired MAT with four-fold titre increase were classified as quality 2. Studies which used other methods for laboratory confirmation were considered quality 3. Statistical analysis for risk factors in the eligible studies included multivariable logistic regression (quality 1) or only unadjusted logistic regression (quality 2). If confidence intervals or equivalent measures of the precision of the estimates were not specified, the manuscript was graded as quality 3. The final quality score was taken as the lower of the two assessments. Due to the small number of eligible studies, all three quality levels were included in the final analysis.

### Data analysis

A descriptive data analysis was performed using the statistical software environment R v 3.2 package to calculate the medians (median of log transformed odds ratios) in order to avoid the impact of outliers. Initial inspection revealed considerable heterogeneity of the risk factors among studies. Therefore, studies were not weighted according to sample size. Additional analysis included risk factors stratified by geographic regions, study type and study population.

## Results

**Fig 1:**
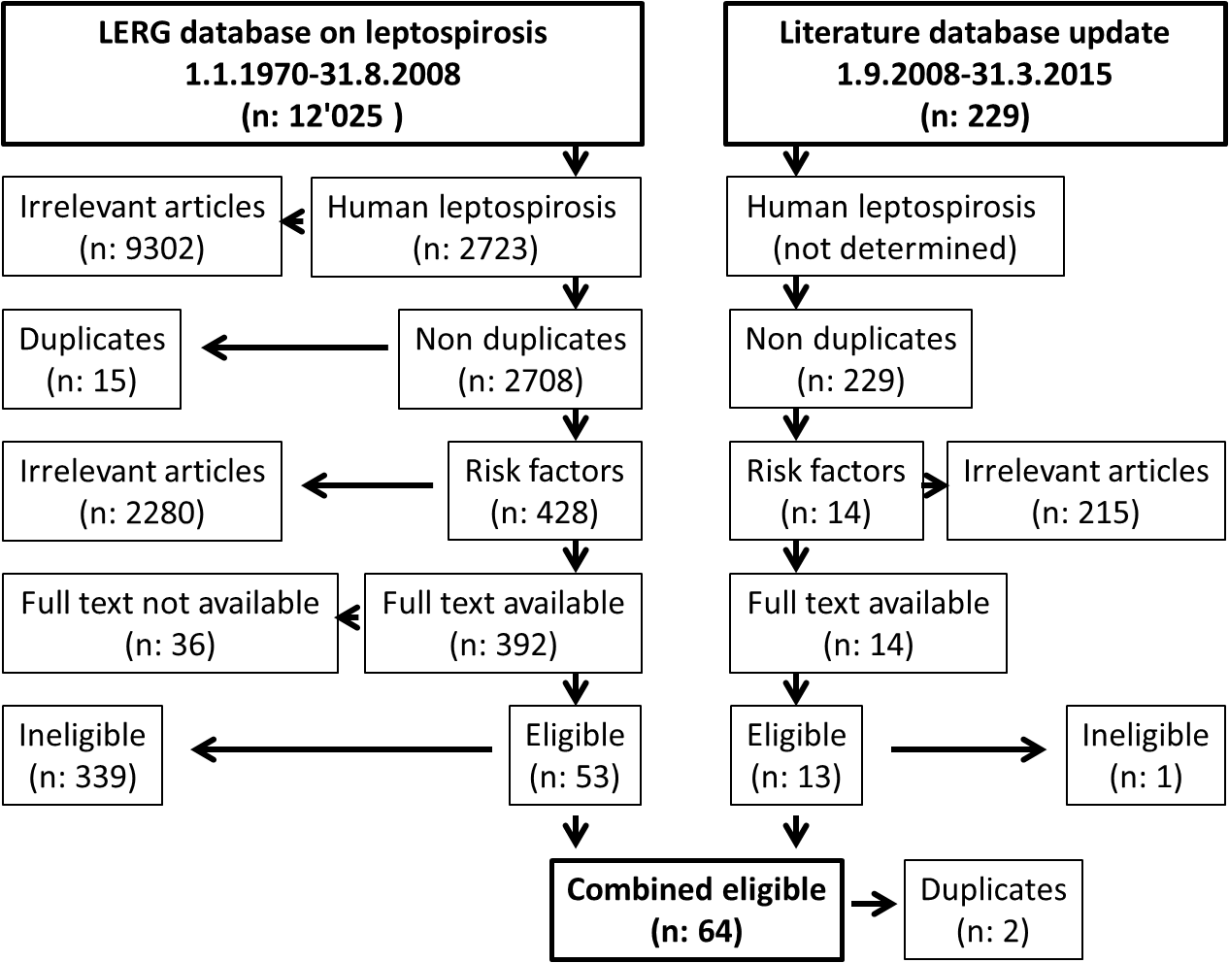
Flowchart of systematic review and identification of articles.

There were 2723 studies in the initial database, 428 of which were related to leptospirosis risk factors (Fig 1, S2 Fig). Out of 392 studies for which the full text was available, there were 53 articles which fulfilled the eligibility criteria and were included in the analysis. The text mining algorithm identified additional 229 potentially relevant articles published between 1.9.2008 and 31.3.2015. Of those 13 were considered eligible and were included in the review. Two articles were identified in both searches because the electronic version was published before and the print issue was published after September 2008. Therefore, we included in total 64 articles. Only one study was graded as highest quality (grade 1). An additional 11 studies were judged as quality grade 2. Due to the low number of studies meeting the highest quality level, stratification by study quality to assess bias was not possible.

### Geographic distribution and study characteristics of eligible studies

**Fig 2:**
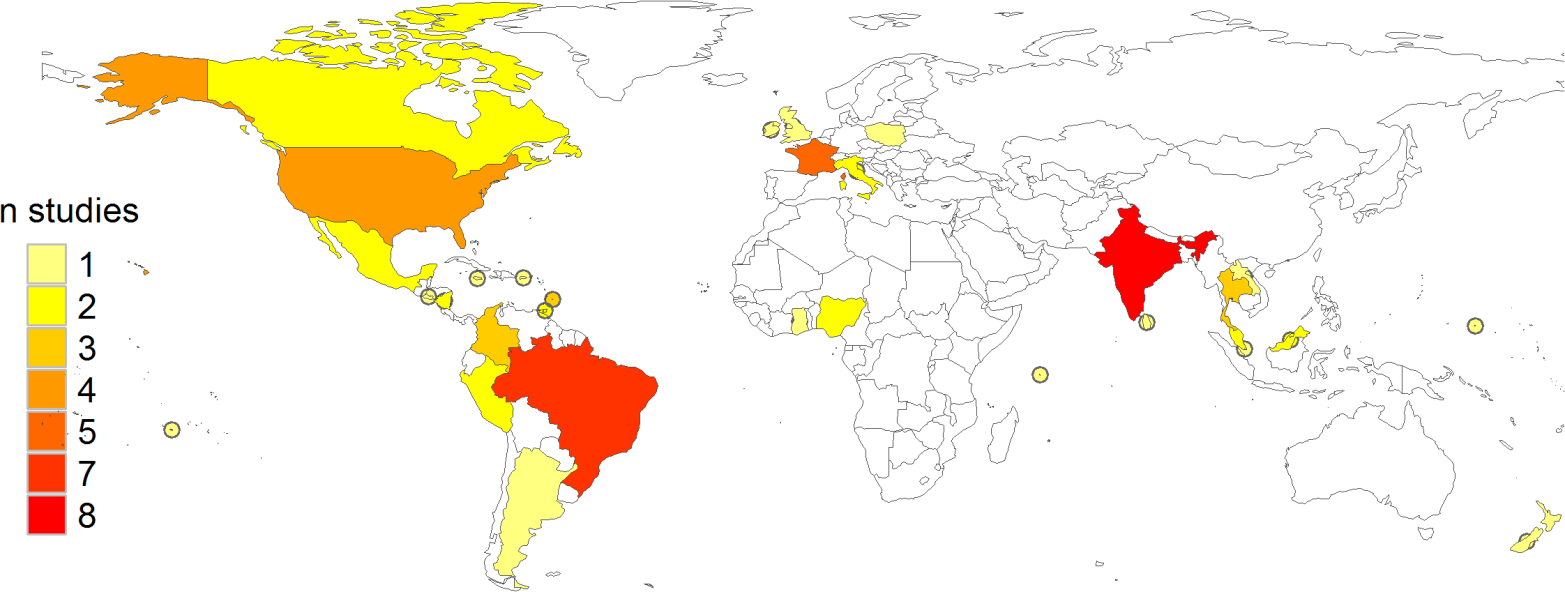
Number of eligible studies stratified by country.

Of the 64 eligible studies, 12 were conducted in Europe and North America, 19 were from Central and South America, 14 were carried out in Southeast Asia, 16 covered small island ecosystems in the Caribbean, Pacific and Indian Oceans and 3 were conducted in Africa (Fig 2). The countries with the highest number of eligible studies were India (8) and Brazil (7), although more than half of the latter were conducted on the Andaman and Nicobar Islands. Out of the five studies conducted in France, four were done in overseas departments. Thirty-two of the included studies (60%) were published between 2000 and 2009.

The most common study type was cross-sectional (41/64) (Table 1). Nineteen studies followed a case control design. The remaining four studies determined risk factors using a retrospective cohort study design, a prospective cohort study design, geospatial modelling and routine data, respectively. Forty-four studies were field-based surveys and 20 were conducted in hospitals or other health facilities.

**Table 1:** Data extraction table.

Year	Country	First author	Confirmed	Suspected	Sample size	Study type	Study population
1979	Barbados	Damude	45	115	561	cross sectional	patient
1983	USA	Demers	50	-	237	cross sectional	general
1985	Trinidad	Everard	328	654	1375	cross sectional	general
1985	UK	Gill	4	257	257	cross sectional	general
1985	USA	Demers	21	32	285	cross sectional	general
1986	Ghana	Hogerzeil	153	460	460	cross sectional	general
1987	Italy	Cacciapuoti	48	-	660	cross sectional	patient
1987	Singapore	Chan	40	300	300	case-control	general
1989	Trinidad	Everard	53	558	1000	longitudinal	general
1991	Nigeria	Ezeh	128	710	710	cross sectional	general
1992	Brazil	Vasconcelos	59	208	208	cross sectional	general
1992	USA	Childs	31	-	1150	cross sectional	general
1994	Italy	Cacciapuoti	305	-	2534	cross sectional	patient
1995	Barbados	Everard	398	816	816	cross-sectional	patient
1995	Canada	DeSerres	9	76	76	cross sectional	general
1996	Martinique	Lhomme	48	518	518	cross sectional	patient
1997	Barbados	Doughlin	22	-	60	case-control	general
1998	India	Murhekar	550	1014	1014	case-control	general
1998	Nicaragua	Trevejo	61	2259	2259	case-control	patient
1999	Brazil	Almeida	34	149	386	case-control	general
1999	Seychelles	Bovet	75	125	250	case-control	general
2000	Guadalupe	Nardone	64	124	124	case-control	patient
2000	Nicaragua	Ashford	84	566	566	cross sectional	general
2000	Thailand	Tangkanakakul	59	80	177	case-control	patient
2001	Brazil	Barcellos	87	-	87	other	general
2002	Brazil	Sarkar	66	157	226	case-control	patient
2002	India	Natarajaseenivasa	225	329	329	cross sectional	general
2002	Mexico	Vado-Solis	57	400	400	cross sectional	general
2002	Peru	Cruz	115	457	457	cross sectional	general
2002	Thailand	Phraisuwan	43	104	104	case-control	general
2002	USA	Morgan	64	389	834	cross sectional	general
2003	El Salvador	Silva	39	73	73	cross sectional	general
2003	India	Karande	18	53	53	cross sectional	patient
2003	Ireland	Boland	6	62	62	cross sectional	general
2003	Mexico	Leal-Castellanos	441	1169	1169	cross sectional	general
2003	Peru	Cespedes	26	71	71	cross sectional	patient
2004	Reunion	Rachou	36	78	78	case-control	general
2004	India	Vijayachari	352	1253	1544	cross sectional	general
2004	India	Manocha	25	346	346	cross sectional	general
2004	Malaysia	Koay	30	46	46	case-control	general
2004	Poland	Krawczyk	14	457	457	cross sectional	general
2005	Colombia	Najera	26	334	334	cross sectional	general
2005	Guadalupe	Herrmann-Storck	212	897	897	cross sectional	patient
2005	Puerto Rico	Bruce	42	730	730	case-control	patient
2006	Brazil	Goncalves	6	150	150	cross sectional	general
2006	Brazil	Aguiar	28	276	276	cross sectional	general
2006	India	Sharma	322	611	611	other	general
2007	Guadalupe	Storck	165	488	488	cross sectional	patient
2007	F.Polynesia	Coudert	33	113	113	cross sectional	patient
2008	Argentina	Vanasco	182	812	812	case-control	general
2008	Colombia	Diaz	52	273	273	cross sectional	general
2008	Laos	Kawaguchi	97	-	406	cross sectional	general
2008	India	Bhardwaj	62	129	129	case-control	general
2009	Colombia	Padmanabha	228	642	642	cross sectional	general
2009	India	Sugunan	52	114	52	case-control	patient
2010	Jamaica	Keenan	43	77	77	case-control	patient
2012	Canada	Sampassa-Kanyinga	60	264	264	cross sectional	general
2012	Thailand	Chusri	150	11	150	cross sectional	general
2013	Malaysia	Rafizah	84	999	999	cross sectional	patient
2013	Kenya	Awosanya	15	15	30	case-control	general
2014	Brazil	Felzemburgh	51	1585	1585	cohort	general
2014	Micronesia	Colt	11	54	54	cross sectional	patient
2014	New Zealand	Dreyfus	62	567	567	cross sectional	general
2015	Sri Lanka	Agampodi	112	401	112	case-control	patient

Complete references for these studies are presented in S1 Text

### Water, agriculture and landscape risks

**Fig 3:**
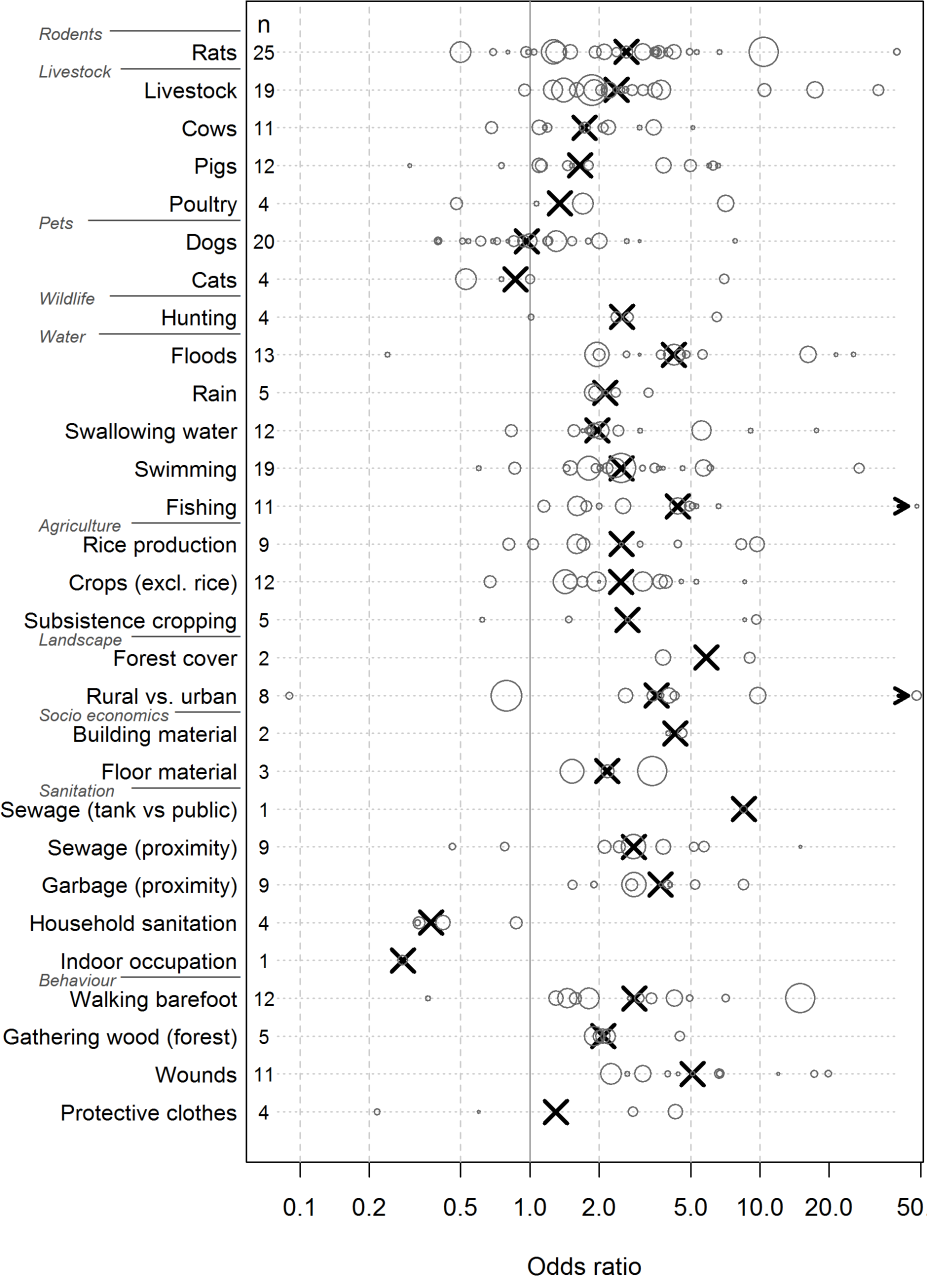
Environmental risk factors for leptospirosis. Circles represent odds ratios of individual studies, with the circle area proportional to the size of the study population. Crosses represent the median odds ratios.

Contact with still water, most often through swimming, was investigated in 19 studies (Fig 3). In 17 of these studies, swimming was related to an increased risk of infection with odds ratios (ORs) ranging from 1.5 to 87.0. It is important to note that the highest OR (87.0) was found in a study that implemented a case control design within an outbreak investigation, which, therefore, lacks external validity. The second highest OR (27.0) was found in farmers in Brazil but was based on a very small sample size (n = 15). Out of the 2 studies which found no association with water one was conducted in Lao PDR. In this study, neither collecting water from streams nor swimming in streams were related to risk of infection (OR: 0.8 and 0.9, respectively) [11]. The other was conducted in Jamaica. Participating in fresh water activities was associated with a reduced risk (OR: 0.6) but the confidence interval was broad and included unity [12]. Fishing was identified as associated with increased risk in all 11 studies investigating this risk factor. One study recorded a very high OR of 244; however, in this study a sero-epidemiological survey among fish farm workers had been compared with routine data from the general population [13]. Floods and heavy rain were associated with leptospirosis in almost all studies investigating these risk factors (n = 17). Three studies, two of them on islands, reported ORs above 6 indicating that the flood was the direct cause of an epidemic rather than a risk factor. Also in this case the study from Jamaica surprisingly found a reduced risk in persons living in previously flooded homes (OR: 0.24) [12].

Living in rural areas was associated with increased risk of leptospirosis infection in six out of eight studies comparing rural and urban residents. The ORs ranged from 2.0 to 9.8 and appeared unrelated to geographic study location, i.e. whether the study was conducted in a developed country or in a resource poor setting (Fig 3, S1 Text). Only one study, among slaughterhouse personnel, found that workers living in peri-urban or rural zones were at lower risk (OR: 0.1) [14]. However, in this study, the number of people from urban areas was very small (n = 8), and this could possibly be a stochastic artifact. One study from Italy found an OR close to one [15]. Nine studies investigated rice production as a risk factor, with seven in Southeast Asia (five mainland and two islands), one in Peru and one in the Caribbean. Of these, one study from Lao PDR found no association between rice farming and leptospirosis. However, rice farming is the predominant occupation in the study area, making the interpretation difficult. The authors concluded based on differences in the gender ratio that occupational exposure seems to be of minor importance in this setting [11]. A study from Trinidad also found no relationship, with ORs close to one [16]. The remaining seven studies identified rice production as a risk factor with ORs ranging from 1.6 to 9.7. Other crops were identified as risk factors in nine of ten studies with ORs between 1.4 and 8.6. Again in this case, the study from Lao PDR came to the opposite conclusion (OR 0.7).

### Social, sanitation and behavioural risks

Factors considered as associated with poor living conditions included dirt floors, proximity to sewage and lack of sanitation, in addition to behavioural factors such as walking barefoot, uncovered wounds and collecting firewood (Fig 3, S1 Text). Eleven out of twelve studies identified walking barefoot as a risk factor. Only the study conducted in Jamaica found an OR below 1 (0.36) [12]. Further, proximity to open sewers was found to be associated with increased risk of infection in six out of eight studies. The other two studies were from India and Brazil [17,18]. Due to the small number of studies investigating the other risk factors the odds ratios should be interpreted with care. For instance, in the case of good household sanitation, shown in four studies, all suggested a protective effect. All studies described above were carried out in resource poor settings. Interestingly, one study in New Zealand observed an elevated risk associated with wearing of protective clothes among abattoir workers [19]. However, this effect was only observed for safety glasses in one subgroup, whereas the impact of protective gloves was not been reported. With the exception of wounds (n = 2) and protective clothes (n = 1), no social, sanitation or behavioural risks factor have been investigated in Europe or North America.

### Animals as risk factors

Twenty-five studies evaluated exposure to rodents, most commonly the genus *Rattus*, but the definition of exposure varied broadly. A majority of the studies were done in South America (10 studies) and on islands (8 studies). Rodents play a major role in transmission (median OR 2.6), but there was considerable variation among the studies, with ORs ranging from 0.5 to 39.5. The highest OR was found in a study conducted in (mainland) France. The smallest effect was found in an urban setting, where the authors noted that the variable specified as “previous exposure to rat excrement” might measure awareness rather than the actual contact. Livestock production was investigated in 18 studies and was associated with increased risk of infection, with the exception of one study in Laos. Notably, a more representative survey conducted in Italy by the same author several years later identified animal contact as an important risk factor. Cattle (10 studies) and pig (11 studies) production were associated with increased risk in most studies. A study from Micronesia observed an OR of 0.3 associated with presence of pigs and also neither dogs nor rats were associated with leptospirosis cases. The authors presumed that lack of variation in the exposure has contributed to these spurious findings because within the study area these animals are found near most homes [20]. In total, 19 studies evaluated the impact of dog ownership on leptospirosis risk. The median OR was close to one, indicating that dogs are not a risk factor in many settings. Surprisingly, four studies found very low risk factors of about 0.5 indicating protective effects. However, sample sizes in two studies were small and the estimates were not statistically significant. In the third study, the authors pointed out that only the univariate analysis showed a statistical significant effect, which disappeared in the multivariate model, concluding that confounding was the most likely explanation. One study in Nicaragua implemented a case control design after an epidemic that followed heavy flooding. The high prevalence among dogs at households from human leptospirosis cases, as well as the identified *Leptospira* strains, suggested that the epidemic likely resulted from exposure to flood water which was contaminated by urine from infected dogs [21]. A study conducted in Peru postulated that dogs are one of the main transmission reservoirs between man and wildlife reservoirs [22]. Unfortunately, with few exceptions, it was not reported whether the dogs were vaccinated against leptospirosis; therefore, a stratified analysis was not possible. Poultry and cats were evaluated in four studies each. A consistent pattern could not be observed. Noticeably, a strong association was found between cats in the home and acute leptospirosis in one study. The authors pointed out that this association may not be causal as people in the Seychelles often keep cats to control rat populations around the home [23]. Surprisingly, only two studies investigated the impact of rodents on mainland Asia.

### Risk factors in Africa

Since information on leptospirosis in Africa is scarce, we included all identified studies from this continent. However, only three eligible studies were conducted in Africa. A study in Ghana assessed agriculture (cocoa farming) and from the two conducted in Nigeria one considered the risk of disease in abattoir workers and one in kennel workers. In all cases, the investigated activities were seen to increase risk of leptospirosis transmission.

### Risk factors stratified by study type and study population

In general, the ORs were higher in case control/cohort studies as compared to cross-sectional studies, while field-based studies were associated with lower OR compared to hospital-based studies (S1 Text).

### Serovars and serogroups by continent

Occurrence and distribution of *Leptospira* serovars could indicate possible transmission sources. As pointed out by several authors, because of cross-reactivity, these findings should be interpreted carefully. In addition, different studies used various batteries of antigens in the MAT and, most importantly, serogroups are often unrelated to taxonomy and host species. Out of the 53 studies in the initial review, 21 reported the identified serovars and 32 reported the serogroups. In South America, the serogroups showed a higher diversity compared to other regions (S1 Fig). In contrast, island ecosystems more often showed one dominant serogroup. The serovar Bim (serogroup Autumnalis) was frequently found in Barbados.

## Discussion

This review confirms the complex environmental transmission pathways of leptospirosis, as summarised in recent reviews [3]^,^[6] and textbooks [5].

Factors related to socio-economics, sanitation and risky behaviour showed a consistent pattern of being associated with increased leptospirosis risk in all settings. However, with only two exceptions (presence of wounds and protective clothes), none of these factors have been investigated in developed countries. The interpretation is compromised by the small number of studies investigating each risk factor. Many studies consistently reported high risk from water related exposures. Floods and rain can be considered as one of the main risk factors in tropical countries. This finding is consistent with a recent review conducted by Lau and colleagues [24]. In addition they hypothesise that due to global warming extreme weather events will occur with increasing frequency and intensity worldwide. Currently, in North America and Europe water related risks are usually related to recreational exposure. As expected, agricultural practices of rice and crop cultivation were connected with leptospirosis risk. Rodents, livestock and pets exhibited a high variation among studies, especially for dogs and cats. For dogs, the heterogeneity might be largely explained by varying levels of vaccination coverage. However, several outbreaks associated with immunised dogs have been reported [1]. These are usually caused by serovars which have been previously rarely reported in dogs, so the antigens are not included in the canine vaccines [25]. It is noteworthy that vaccination protects against the disease but does not completely abolish the carrier state, allowing for continued excretion of leptospire serovars [26]. The role of cats is controversial in the literature, with cats both reported to facilitate the infection as well as to play a protective role in different studies. It has been postulated that cats have strong resistance to leptospires, since they often eat rodents. In addition, feline urine is acidic, which could potentially decrease viability of excreted leptospires. However, the role of cats as a source of infection has recently gained more attention [27]. Due to the small number of studies on hunting, the results which generally show a risk of leptospirosis should be interpreted with care. Wildlife might play an important role in the transmission pathway. Clinical disease in wild animals appears to be less severe than that described in subsequently infected humans. In animals, the course of disease is often short, although it can be associated with sequelae and an asymptomatic carrier state. The wide range of animals which can serve as an infection source for leptospirosis includes primarily mammals, making leptospirosis one of the most geographically widespread zoonotic diseases [1],[28]. It is important to note that, although numerous pathogenic serogroups of leptospires exist, not all exhibit the same virulence in each animal species, as evidenced, for instance, by greater disease frequency caused by *L*. *interrogans*, serovar canicola in dogs [29].

Eight out of 53 studies mentioned rural versus urban areas as a risk factor. Although rural areas may be at higher risk, leptospires in urban environments may be more virulent [30]. In addition, urban leptospirosis is often associated with epidemics and is possibly underrepresented in this review because outbreak reports were excluded. It has also been noted that the increasing pressures on wildlife agglomerations at the peri-urban interface might lead to increased risk of leptospirosis in urban areas in the future [25].

Because of some observed high heterogeneity, it is difficult to translate these presented results into general recommendations for designing effective healthcare interventions. However, detailed analysis in specific geo-climatic contexts could further delineate this finding and provide more focused recommendations. Similarly, the relative importance of the transmission pathways might be modified accordingly to emphasise soil and water related exposures over rodent and animal exposures. A unique feature of spirochetes allows for survival of most pathogenic *Leptospira* serovars outside a host, consequently contributing to maintain infection cycles in reservoirs [26]. Future attempts to develop leptospirosis transmission models should primarily address environmental water related exposures as a main driver for transmission. Such an approach would be mathematically more tractable and could include constant, seasonal and rare event (e.g., typhoon) components. The underlying rodent population dynamic feeds environmental contamination, but comprehensive inclusion would require more advanced epidemiological studies to include a better understanding of setting specific rodent population dynamics. Future epidemiological studies should address ecological, climatic and rodent demographic components for a more detailed understanding of environmental contamination. The primary, universal motivation for the majority of models is a better understanding of the dynamics of infection and prediction of impact of interventions, such as vaccines [31].

A substantial amount of presumptive data (62/339 ineligible studies) was excluded from this analysis. In most cases, the exclusion was based on inadequate study design; specifically, these were prevalence and incidence studies, national data, outbreak investigations and case series. Some studies lacked sufficient descriptive detail for evaluation. A brief summary of the existing presumptive risk data complements our findings from the included, well defined epidemiological studies. Of the excluded presumptive studies, there were 23 in Europe or North America, 12 in Central or South America, 14 in Asia,7 in island eco-systems, 3 in Australia and 3 in Africa. The risk factors described most often were related to exposure to contaminated water, especially flooding and high rainfall. Agricultural exposures, both crop and livestock, were also frequently cited. Exposures related to poor sanitation, e.g. sewage and garbage, rodents and dogs were frequently noted, while low altitude was mentioned in only two studies.

In addition to the limited number of studies available, there is great variability in the definition of risk factors between studies. The definition of exposure to rodents, for example, ranged from rodent control workers [32], past exposure to rodent excrement [33] to sighting groups of more than five rats [34], and sometimes this risk factor lacked a clear description. This might partially explain the high heterogeneity among studies.

Another limitation is that many authors tend to provide only estimates for risk factors which are statistically significant. Likewise, for statistical reasons, multivariate logistic regression models are usually performed after a variable subset selection procedure which identifies variables with high predictive power and, therefore, high odds ratios. For this reason, odds ratios close to one might have been omitted in many cases, leading to upward biased medians. In addition, studies with small sample sizes and no significant effects are less likely to be published; however, a formal assessment of the publication bias was constrained by the substantial heterogeneity of estimates. The study designs and risk characteristics in the included studies are very heterogeneous, resulting in comparisons and groupings which might not always be appropriate. Future effort to harmonise exposure definitions and study designs would greatly enhance the comparability. There was often a focus on either a patient population or the general population, although sometimes a combination of both. For instance, one case control study selected patients as cases while the controls were healthy neighbours from the same village. In instances where study design followed an outbreak, the resulting OR was higher in comparison to other studies. Moreover, quality differed in individual studies, where some clearly mentioned diagnostic tests used, but others did not necessitating reviewer conjecture based on the available data.

The presented results need to be interpreted carefully due to the low number of fundamentally sound epidemiological studies. Relatively few studies met stringent epidemiological and diagnostic criteria, for instance not differentiating between current or past disease because clinical signs and symptoms were not investigated. Such studies were considered not eligible. On the other hand, signs and symptoms are highly variable and not easily summarized. Therefore, it remains unclear if studies reporting signs and symptoms should be considered per se to be of higher quality. However, repeated exposure—e.g., slaughterhouse personnel—might induce seroconversion without infection, thereby resulting in a protective effect.

The time frame for inclusion of this analysis was limited to the consensus database of LERG, but it would not be expected that the environmental risk factors would have changed significantly since 2009.Future work on risk factors for leptospirosis in endemic settings should meet the following minimal criteria:

1. Community based cohort studies with appropriate sample size calculation. If cohort designs are not feasible due to budget or time constraints, properly designed prospective case-control studies could be an alternative. Controls should preferably be selected from matching community members.
2. Clearly defined exposure variables.
3. Clear leptospirosis case definition including clinical signs/symptoms in addition to laboratory confirmation.
4. Laboratory confirmation by culture of *Leptospira* spp., MAT, PCR, or ELISA, provided these received local validation and/or are associated to international proficiency testing schemes.
5. Appropriate statistical analysis.
6. If appropriate, identification of suspected animal risks—analogous to Bonfoh et al.[35]—following a “one health” study approach, allows for identification of the source of transmission.

Future work should also improve the characterisation of risk groups and their specific exposure, as, for instance, low income rural and urban dwellers, occupational exposure, leisure and recreational exposure in relation to environmental risks. Future transmission models based on such data may then be used as a tool for the estimation of disease burden and comparative analyses of interventions within and outside the public health sector.

## Supporting Information

S1 Checklist Prisma checklist. (PDF)

S1 Text Full references of eligible studies and risk-factors stratified by geography, study type and population. (PDF)

S2 Text Details on the text mining algorithm to update the articles in the systematic review. (PDF)

S1 Fig Serogroups reported in 32 studies. (PDF)

S2 Fig Prisma flowchart. (PDF)
