# Supplementary material for: Environmental and Behavioural Determinants of Leptospirosis Transmission: A Systematic Review
Source: PLoS Negl Trop Dis. 2015 Sep 17;9(9):e0003843. doi: 10.1371/journal.pntd.0003843 (PMC4574979; doi:10.1371/journal.pntd.0003843)

Supplementary web material 1 - Environmental and Behavioural Determinants of Leptospirosis Transmission: A systematic review

Appendix 1: References for eligible studies 2

Appendix 2: Risk factors on water, agriculture and landscape by geographic regions 7

Appendix 3: Risk factors concerning socio economics, sanitation and behaviour stratified by geographic locations 8

Appendix 4: Animal risk factors stratified by geographic locations 9

Appendix 5: Risk factors stratified by study type 10

Appendix 6: Risk factors stratified by study population 11

Appendix 1: References for eligible studies

Agampodi, S.B., Nugegoda, D.B., Thevanesam, V., Vinetz, J.M., 2015. Characteristics of rural leptospirosis patients admitted to referral hospitals during the 2008 leptospirosis outbreak in Sri Lanka: implications for developing public health control measures. Am. J. Trop. Med. Hyg. 92, 139–144. doi:10.4269/ajtmh.14-0465

Aguiar, D., Cavalcante, G., Camargo, L., Labruna, M., Vasconcellos, S., Souza, G., Gennari, S., 2007. Anti-Leptospira Spp And Anti-Brucella Spp Antibodies In Humans From Rural Area Of Monte Negro Municipality , State Of Rondônia , Brazilian Western Amazon. Brazilian Journal of Microbiology 38, 93–96.

Almeida, L.P. de, Martins, L.F. da S., Brod, C.S., 1999. Fatores de risco associados à presença de anticorpos antileptospira em trabalhadores do serviço de saneamento ambiental. Ciência Rural 29, 511–516.

Ashford, D.A., Kaiser, R.M., Spiegel, R.A., Perkins, B.A., Weyant, R.S., Bragg, S.L., Plikaytis, B., Jarquin, C., De Lose Reyes, J.O., Amador, J.J., 2000. Asymptomatic infection and risk factors for leptospirosis in Nicaragua. The American Journal of Tropical Medicine and Hygiene 63, 249–254.

Awosanya, E.J., Nguku, P., Oyemakinde, A., Omobowale, O., 2013. Factors associated with probable cluster of leptospirosis among kennel workers in Abuja, Nigeria. Pan Afr Med J 16, 144. doi:10.11604/pamj.2013.16.144.3529

Barcellos, C., Sabroza, P.C., 2001. The place behind the case: leptospirosis risks and associated environmental conditions in a flood-related outbreak in Rio de Janeiro. Cad Saude Publica 17 Suppl, 59–67.

Bhardwaj, P., Kosambiya, J.K., Desai, V.K., 2008. A case control study to explore the risk factors for acquisition of leptospirosis in Surat city, after flood. Indian J Med Sci 62, 431–438.

Boland, M., Sayers, G., Coleman, T., Bergin, C., Sheehan, N., Creamer, E., O’Connell, M., Jones, L., Zochowski, W., 2004. A cluster of leptospirosis cases in canoeists following a competition on the River Liffey. Epidemiol. Infect. 132, 195–200.

Bovet, P., Yersin, C., Merien, F., Davis, C.E., Perolat, P., 1999. Factors associated with clinical leptospirosis: a population-based case-control study in the Seychelles (Indian Ocean). Int J Epidemiol 28, 583–590.

Bruce, M.G., Sanders, E.J., Leake, J.A.D., Zaidel, O., Bragg, S.L., Aye, T., Shutt, K.A., Deseda, C.C., Rigau-Perez, J.G., Tappero, J.W., Perkins, B.A., Spiegel, R.A., Ashford, D.A., 2005. Leptospirosis among patients presenting with dengue-like illness in Puerto Rico. Acta Tropica 96, 36–46. doi:10.1016/j.actatropica.2005.07.001

Cacciapuoti, B., Ciceroni, L., Pinto, A., Apollini, M., Rondinella, V., Bonomi, U., Benedetti, E., Cinco, M., Dessì, S., Dettori, G., 1994. Survey on the prevalence of leptospira infections in the Italian population. Eur. J. Epidemiol. 10, 173–180.

Cacciapuoti, B., Vellucci, A., Ciceroni, L., Pinto, A., Taggi, F., 1987. Prevalence of leptospirosis in man. Pilot survey. Eur. J. Epidemiol. 3, 137–142.

Céspedes, M., Ormaeche, M., Condori, P., Balda, L., Glenny, M., 2003. Prevalencia de leptospirosis y factores de riesgo en personas con antecedentes de fiebre en la provincia de Manu, Madre de Dios, Perú. Rev Peru Med Exp Salud Publica 20, 180–185.

Chan, O.Y., Chia, S.E., Nadarajah, N., Sng, E.H., 1987. Leptospirosis risk in public cleansing and sewer workers. Ann. Acad. Med. Singap. 16, 586–590.

Childs, J.E., Schwartz, B.S., Ksiazek, T.G., Graham, R.R., LeDuc, J.W., Glass, G.E., 1992. Risk factors associated with antibodies to leptospires in inner-city residents of Baltimore: a protective role for cats. Am J Public Health 82, 597–599.

Chusri, S., Sritrairatchai, S., Hortiwahul, T., Charoenmak, B., Silpapojakul, K., 2012. Leptospirosis among river water rafters in Satoon, southern Thailand. J Med Assoc Thai 95, 874–877.

Colt, S., Pavlin, B.I., Kool, J.L., Johnson, E., McCool, J.P., Woodward, A.J., 2014. Human leptospirosis in The Federated States of Micronesia: a hospital-based febrile illness survey. BMC Infect. Dis. 14, 186. doi:10.1186/1471-2334-14-186

Coudert, C., Beau, F., Berlioz-Arthaud, A., Melix, G., Devaud, F., Boyeau, E., Jaomeau, C., Lablee, P., Jarno, P., 2007. [Human leptospirosis in French Polynesia. Epidemiological, clinical and bacteriological features]. Med Trop (Mars) 67, 137–144.

Cruz M., R., Fernández V., F., Arévalo R., H., 2002. Hiperendemicidad de Leptospirosis y factores de riesgo asociados en localidades arroceras del departamento de San Martín - Perú. Rev Peru Med Exp Salud Pública 19, 10–16.

Damude, D.F., Jones, C.J., Myers, D.M., 1979. A study of leptospirosis among animals in Barbados W.I. Trans. R. Soc. Trop. Med. Hyg. 73, 161–168.

De Serres, G., Levesque, B., Higgins, R., Major, M., Laliberté, D., Boulianne, N., Duval, B., 1995. Need for vaccination of sewer workers against leptospirosis and hepatitis A. Occup Environ Med 52, 505–507.

De Vasconcelos, L.M., Ramos-Vieira M das, N., Osório-Cisalpino, E., Cota-Koury, M., 1993. [Survey of anti-Leptospira agglutinins in workers from the city of Londrina-Paraná, Brazil]. Rev. Latinoam. Microbiol. 35, 153–157.

Demers, R.Y., Frank, R., Demers, P., Clay, M., 1985. Leptospiral exposure in Detroit rodent control workers. Am J Public Health 75, 1090–1091.

Demers, R.Y., Thiermann, A., Demers, P., Frank, R., 1983. Exposure to Leptospira icterohaemorrhagiae in inner-city and suburban children: a serologic comparison. J Fam Pract 17, 1007–1011.

Díaz P, L., Zapata, I., Góngora O, A., Parra A, J., Aponte G, L., Gómez L, L., 2008. Detección De Anticuerpos IgM A Leptospira En Humanos En Riesgo Ocupacional En Villavicencio, Meta. Revista MVZ Córdoba 13, 1120–1127.

Douglin, C.P., 1997. Risk Factors for Severe Leptospirosis in the Parish of St. Andrew, Barbados. Emerging Infectious Diseases 3, 78–80. doi:10.3201/eid0301.970114

Dreyfus, A., Benschop, J., Collins-Emerson, J., Wilson, P., Baker, M.G., Heuer, C., 2014. Sero-prevalence and risk factors for leptospirosis in abattoir workers in New Zealand. Int J Environ Res Public Health 11, 1756–1775. doi:10.3390/ijerph110201756

Everard, C.O., Edwards, C.N., Everard, J.D., Carrington, D.G., 1995. A twelve-year study of leptospirosis on Barbados. Eur. J. Epidemiol. 11, 311–320.

Everard, C.O., Hayes, R.J., Edwards, C.N., 1989. Leptospiral infection in school-children from Trinidad and Barbados. Epidemiology and Infection 103, 143–156.

Everard, C.O., Hayes, R.J., Fraser-Chanpong, G.M., 1985. A serosurvey for leptospirosis in Trinidad among urban and rural dwellers and persons occupationally at risk. Trans. R. Soc. Trop. Med. Hyg. 79, 96–105.

Ezeh, A.O., Adesiyun, A.A., Addo, P.B., Ellis, W.A., Makinde, A.A., Bello, C.S., 1991. Serological and cultural examination for human leptospirosis in Plateau State, Nigeria. Cent Afr J Med 37, 11–15.

Felzemburgh, R.D.M., Ribeiro, G.S., Costa, F., Reis, R.B., Hagan, J.E., Melendez, A.X.T.O., Fraga, D., Santana, F.S., Mohr, S., dos Santos, B.L., Silva, A.Q., Santos, A.C., Ravines, R.R., Tassinari, W.S., Carvalho, M.S., Reis, M.G., Ko, A.I., 2014. Prospective study of leptospirosis transmission in an urban slum community: role of poor environment in repeated exposures to the Leptospira agent. PLoS Negl Trop Dis 8, e2927. doi:10.1371/journal.pntd.0002927

Gill, O.N., Coghlan, J.D., Calder, I.M., 1985. The risk of leptospirosis in United Kingdom fish farm workers. Results from a 1981 serological survey. The Journal of Hygiene 94, 81–86.

Gonçalves, D.D., Teles, P.S., Reis, C.R. dos, Lopes, F.M.R., Freire, R.L., Navarro, I.T., Alves, L.A., Muller, E.E., Freitas, J.C. de, 2006. Seroepidemiology and occupational and environmental variables for leptospirosis, brucellosis and toxoplasmosis in slaughterhouse workers in the Paraná State, Brazil. Revista do Instituto de Medicina Tropical de São Paulo 48, 135–140. doi:10.1590/S0036-46652006000300004

Herrmann-Storck, C., Brioudes, A., Quirin, R., Deloumeaux, J., Lamaury, I., Nicolas, M., Postic, D., Perez, J.M., 2005. Retrospective review of leptospirosis in Guadeloupe, French West Indies 1994-2001. West Indian Med J 54, 42–46.

Hogerzeil, H.V., Terpstra, W.J., De Geus, A., Korver, H., 1986. Leptospirosis in rural Ghana. Trop Geogr Med 38, 162–166.

Karande, S., Bhatt, M., Kelkar, A., Kulkarni, M., De, A., Varaiya, A., 2003. An observational study to detect leptospirosis in Mumbai, India, 2000. Archives of Disease in Childhood 88, 1070–1075. doi:10.1136/adc.88.12.1070

Kawaguchi, L., Sengkeopraseuth, B., Tsuyuoka, R., Koizumi, N., Akashi, H., Vongphrachanh, P., Watanabe, H., Aoyama, A., 2008. Seroprevalence of leptospirosis and risk factor analysis in flood-prone rural areas in Lao PDR. Am. J. Trop. Med. Hyg. 78, 957–961.

Keenan, J., Ervin, G., Aung, M., McGwin, G., Jolly, P., 2010. Risk factors for clinical leptospirosis from Western Jamaica. Am. J. Trop. Med. Hyg. 83, 633–636. doi:10.4269/ajtmh.2010.09-0609

Koay, T.K., Nirmal, S., Noitie, L., Tan, E., 2004. An epidemiological investigation of an outbreak of leptospirosis associated with swimming, Beaufort, Sabah. Med. J. Malaysia 59, 455–459.

Krawczyk, M., 2004. [Estimation of transmission hazard of Leptospira Sp. infections in 2 groups of people]. Przegl Epidemiol 58, 207–212.

Leal-Castellanos, C.B., García-Suárez, R., González-Figueroa, E., Fuentes-Allen, J.L., Escobedo-de la Peñal, J., 2003. Risk factors and the prevalence of leptospirosis infection in a rural community of Chiapas, Mexico. Epidemiol. Infect. 131, 1149–1156.

Lhomme, V., Grolier-Bois, L., Jouannelle, J., Elisabeth, L., 1996. Leptospirose en Martinique de 1987 à 1992: bilan d'une étude épidémiologique, clinique et biologique. Med Mal Infect 26, 94–98.

Manocha., H., Ghosha, U., Singh, S., Kishore, J., Ayyagari, A., 2004. Frequency of leptospirosis in patients of acute febrile illness in Uttar Pradesh. Journal of the Association of Physicians India 52, 623–625.

Morgan, J., Bornstein, S.L., Karpati, A.M., Bruce, M., Bolin, C.A., Austin, C.C., Woods, C.W., Lingappa, J., Langkop, C., Davis, B., Graham, D.R., Proctor, M., Ashford, D.A., Bajani, M., Bragg, S.L., Shutt, K., Perkins, B.A., Tappero, J.W., 2002. Outbreak of leptospirosis among triathlon participants and community residents in Springfield, Illinois, 1998. Clin. Infect. Dis. 34, 1593–1599. doi:10.1086/340615

Murhekar, M.V., Sugunan, A.P., Vijayachari, P., Sharma, S., Sehgal, S.C., 1998. Risk factors in the transmission of leptospiral infection. Indian J. Med. Res. 107, 218–223.

Najera, S., Alvis, N., Babilonia, D., Alvarez, L., Mattar, S., 2005. Leptospirosis ocupacional en una región del Caribe colombiano. Salud Publica de Mexico 47, 240–244.

Nardone, A., Campèse, C., Capek, I., 2002. Les facteurs de risques de leptospirose en France métropolitaine Une étude cas-témoin, juillet 1999 - février 2000. Institut de veille sanitaire, Saint-Maurice, France.

Natarajaseenivasan, K., Boopalan, M., Selvanayaki, K., Suresh, S.R., Ratnam, S., 2002. Leptospirosis among rice mill workers of Salem, South India. Jpn. J. Infect. Dis. 55, 170–173.

Padmanabha, H., Hidalgo, M., Valbuena, G., Castaneda, E., Galeano, A., Puerta, H., Cantillo, C., Mantilla, G., 2009. Geographic variation in risk factors for SFG rickettsial and leptospiral exposure in Colombia. Vector Borne Zoonotic Dis. 9, 483–490. doi:10.1089/vbz.2008.0092

Phraisuwan, P., Whitney, E.A.S., Tharmaphornpilas, P., Guharat, S., Thongkamsamut, S., Aresagig, S., Liangphongphanthu, J., Junthima, K., Sokampang, A., Ashford, D.A., 2002. Leptospirosis: skin wounds and control strategies, Thailand, 1999. Emerging Infect. Dis. 8, 1455–1459. doi:10.3201/eid0812.020180

Rachou, E., Ricquebourg, M., Yovanovitch,, J., 2004. La Leptospirose Humaine A La Reunion En 2003 Resultats D'une Etude Cas-Temoins. Observatoire Régional de la Santé, Réunion.

Rafizah, A.A.N., Aziah, B.D., Azwany, Y.N., Imran, M.K., Rusli, A.M., Nazri, S.M., Nikman, A.M., Nabilah, I., Asma', H.S., Zahiruddin, W.M., Zaliha, I., 2013. Risk factors of leptospirosis among febrile hospital admissions in northeastern Malaysia. Prev Med 57 Suppl, S11–13. doi:10.1016/j.ypmed.2012.12.017

Sampasa-Kanyinga, H., Lévesque, B., Anassour-Laouan-Sidi, E., Côté, S., Serhir, B., Ward, B.J., Libman, M.D., Drebot, M.A., Ndao, M., Dewailly, E., 2012. Zoonotic infections in native communities of James Bay, Canada. Vector Borne Zoonotic Dis. 12, 473–481. doi:10.1089/vbz.2011.0739

Sarkar, U., Nascimento, S., Barbosa, R., Martins, R., Nuevo, H., Kalafanos, I., Grunstein, I., Flannery, B., Dias, J., Riley, L., Reis, M., Ko, A., 2002. Population-based case-control investigation of risk factors for leptospirosis during an urban epidemic. American Journal of Tropical Medicine and Hygiene 66, 605–610.

Sharma, S., Vijayachari, P., Sugunan, A.P., Natarajaseenivasan, K., Sehgal, S.C., 2006. Seroprevalence of leptospirosis among high-risk population of Andaman Islands, India. Am. J. Trop. Med. Hyg. 74, 278–283.

Silva, H.R., Tavares-Neto, J., Bina, J.C., Meyer, R., 2003. Leptospirose-infecção e forma subclínica em crianças de Salvador, Bahia. Revista da Sociedade Brasileira de Medicina Tropical 36, 227–233.

Storck, C.H., Postic, D., Lamaury, I., Perez, J.M., 2008. Changes in epidemiology of leptospirosis in 2003--2004, a two El Niño Southern Oscillation period, Guadeloupe archipelago, French West Indies. Epidemiol. Infect. 136, 1407–1415. doi:10.1017/S0950268807000052

Sugunan, A.P., Vijayachari, P., Sharma, S., Roy, S., Manickam, P., Natarajaseenivasan, K., Gupte, M.D., Sehgal, S.C., 2009. Risk factors associated with leptospirosis during an outbreak in Middle Andaman, India. Indian J. Med. Res. 130, 67–73.

Tangkanakul, W., Tharmaphornpil, P., Plikaytis, B.D., Bragg, S., Poonsuksombat, D., Choomkasien, P., Kingnate, D., Ashford, D.A., 2000. Risk factors associated with leptospirosis in northeastern Thailand, 1998. Am. J. Trop. Med. Hyg. 63, 204–208.

Trevejo, R.T., Rigau-Pérez, J.G., Ashford, D.A., McClure, E.M., Jarquín-González, C., Amador, J.J., de los Reyes, J.O., Gonzalez, A., Zaki, S.R., Shieh, W.J., McLean, R.G., Nasci, R.S., Weyant, R.S., Bolin, C.A., Bragg, S.L., Perkins, B.A., Spiegel, R.A., 1998. Epidemic leptospirosis associated with pulmonary hemorrhage-Nicaragua, 1995. J. Infect. Dis. 178, 1457–1463.

Vado-Solís, I., Cárdenas-Marrufo, M.F., Jiménez-Delgadillo, B., Alzina-López, A., Laviada-Molina, H., Suarez-Solís, V., Zavala-Velázquez, J.E., 2002. Clinical-epidemiological study of leptospirosis in humans and reservoirs in Yucatán, México. Rev. Inst. Med. Trop. Sao Paulo 44, 335–340.

Vanasco, N.B., Schmeling, M.F., Lottersberger, J., Costa, F., Ko, A.I., Tarabla, H.D., 2008. Clinical characteristics and risk factors of human leptospirosis in Argentina (1999-2005). Acta Trop. 107, 255–258. doi:10.1016/j.actatropica.2008.06.007

Vijayachari, P., Sugunan, A.P., Murhekar, M.V., Sharma, S., Sehgal, S.C., 2004. Leptospirosis among schoolchildren of the Andaman & Nicobar Islands, India: low levels of morbidity and mortality among pre-exposed children during an epidemic. Epidemiology and Infection 132, 1115–1120.

Appendix 2: Risk factors on water, agriculture and landscape by geographic regions

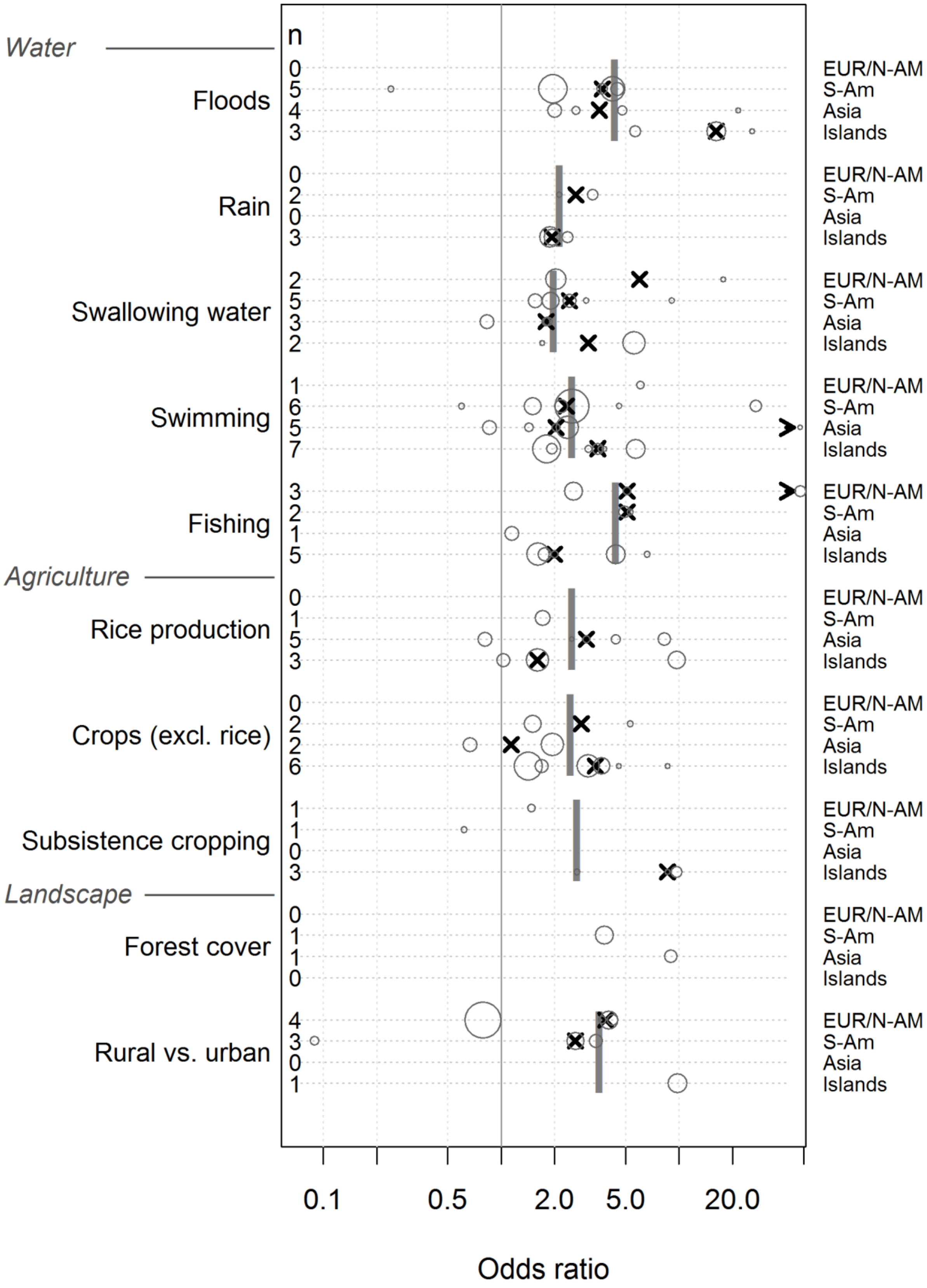

Appendix 3: Risk factors concerning socio economics, sanitation and behaviour stratified by geographic locations

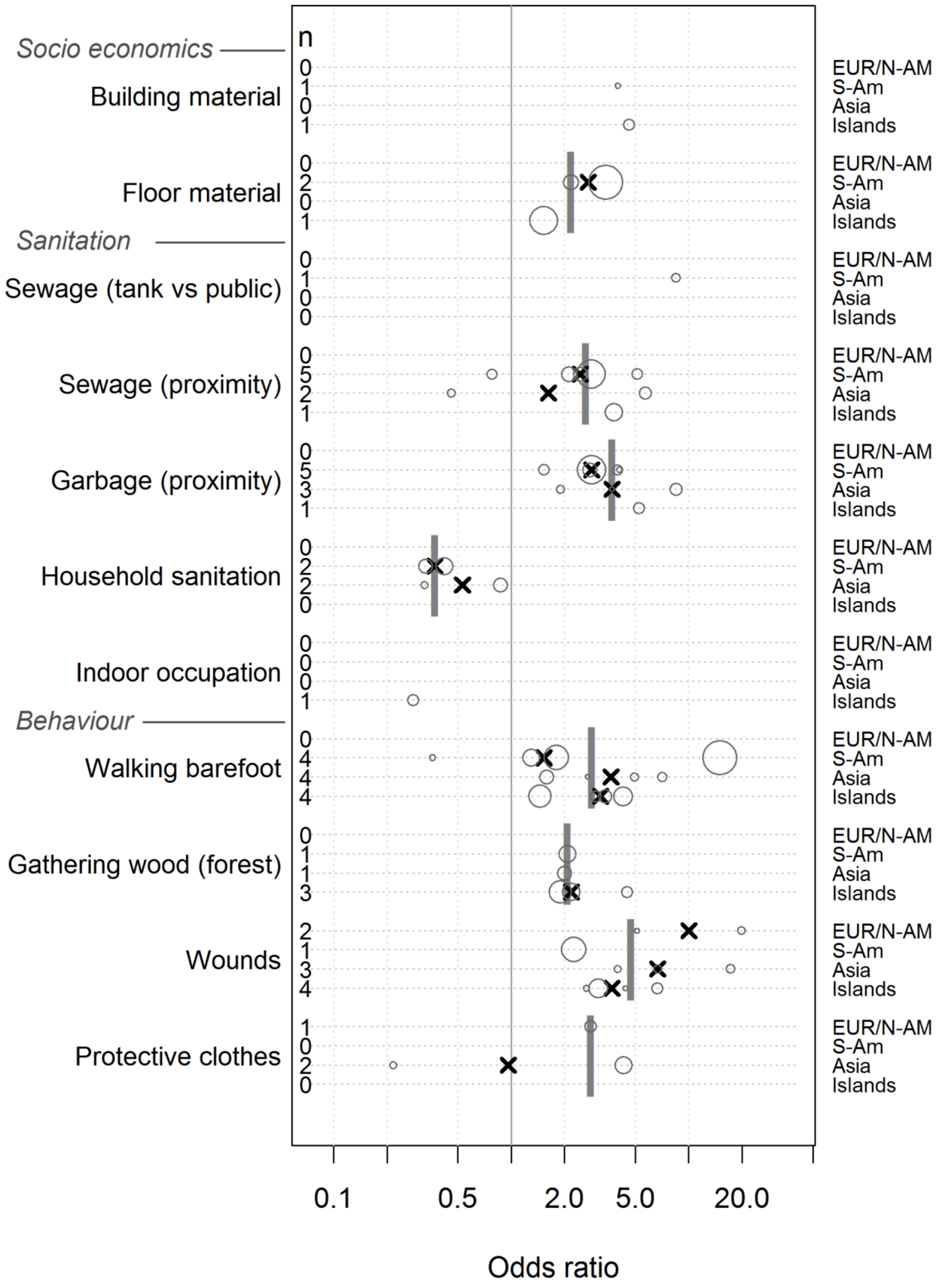

Appendix 4: Animal risk factors stratified by geographic locations

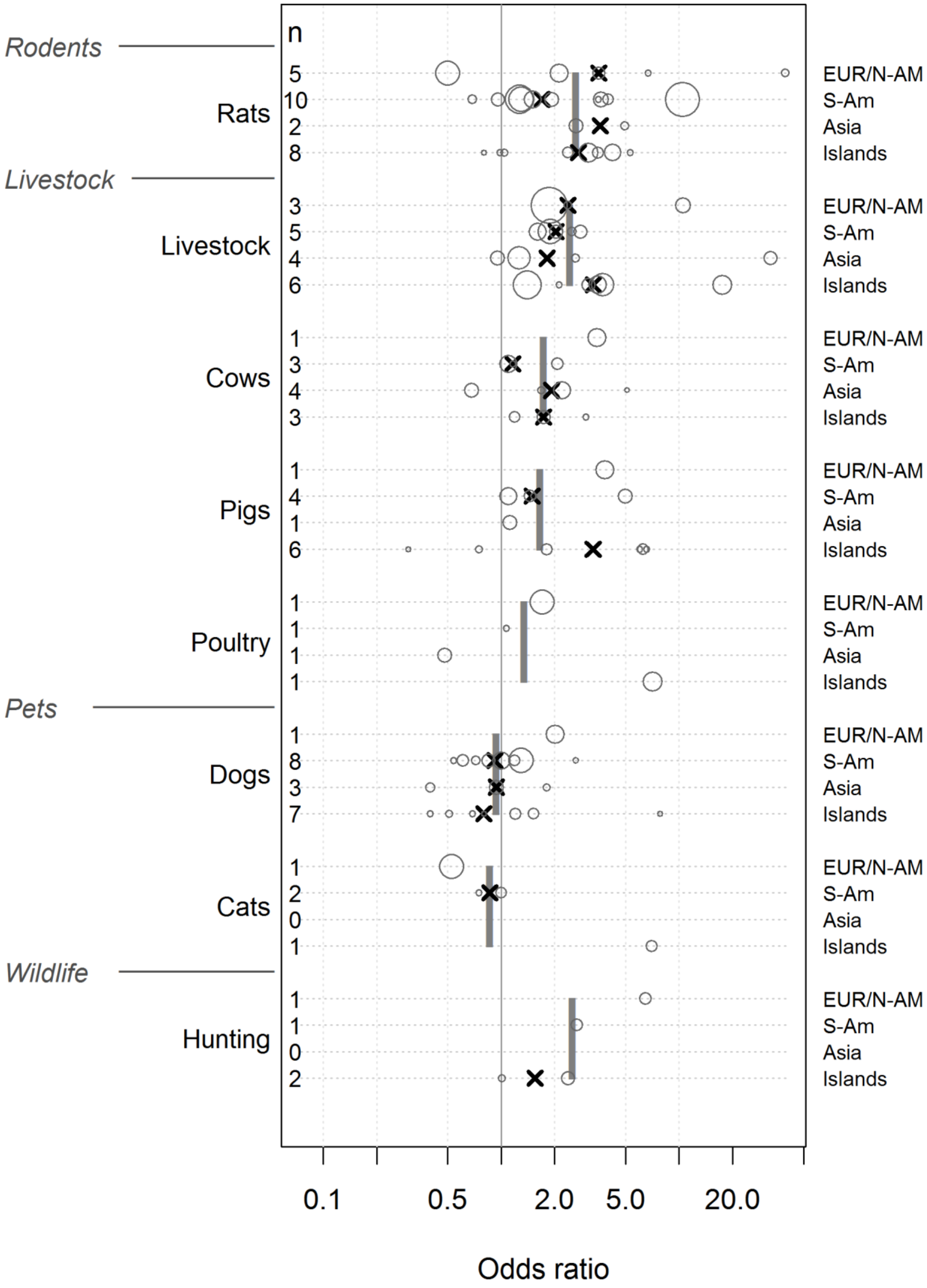

Appendix 5: Risk factors stratified by study type

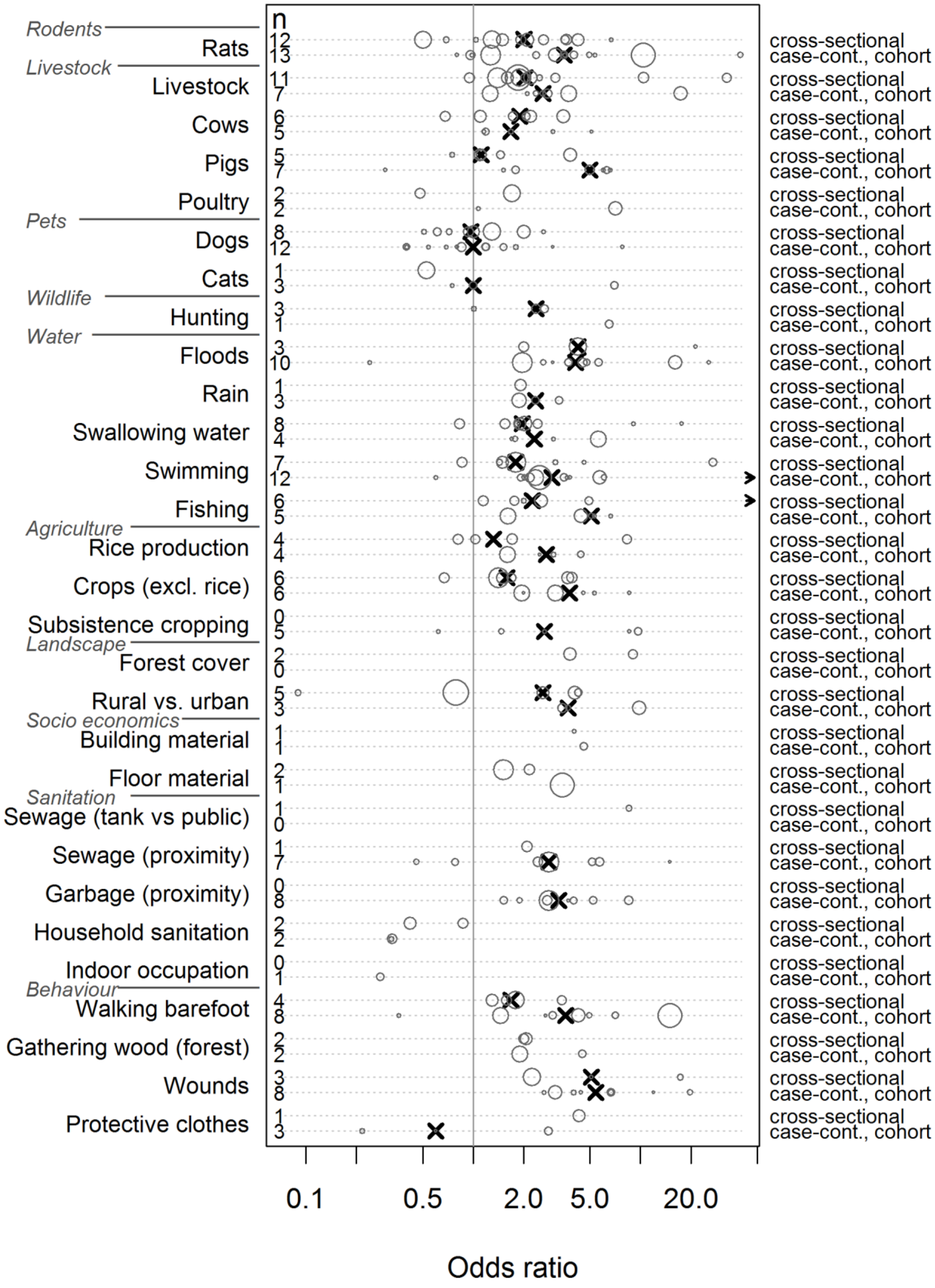

Appendix 6: Risk factors stratified by study population

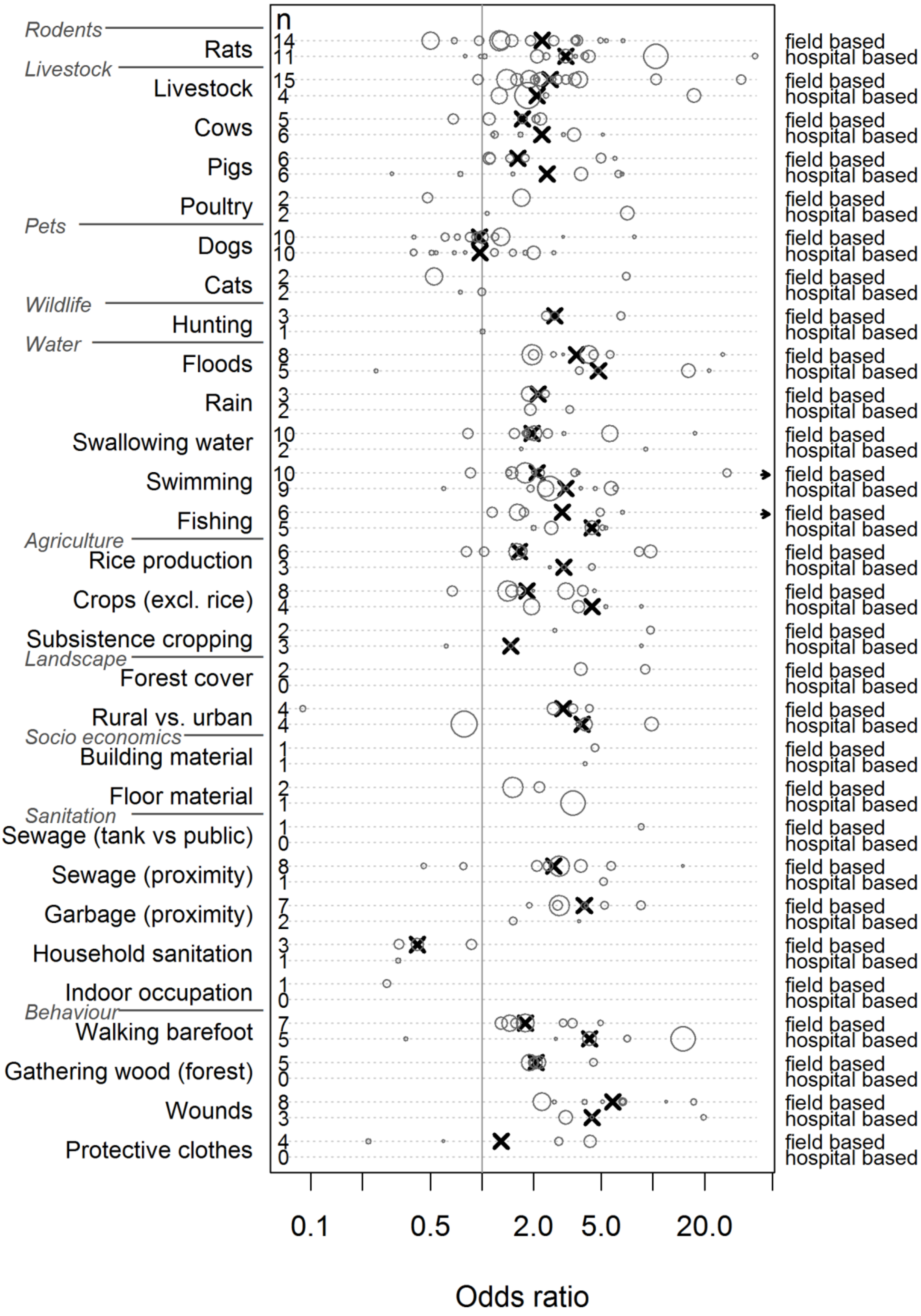

Supplement: S1 Text — (PDF) [file pntd.0003843.s002.pdf]
