# Supplementary material for: Environmental and Behavioural Determinants of Leptospirosis Transmission: A Systematic Review
Source: PLoS Negl Trop Dis. 2015 Sep 17;9(9):e0003843. doi: 10.1371/journal.pntd.0003843 (PMC4574979; doi:10.1371/journal.pntd.0003843)
Supplement: S2 Text — (PDF) [file pntd.0003843.s003.pdf]

Supplementary web material 2 – Text mining Markov Chain algorithm to update the literature search.

The literature database provided by LERG for this review consisted of 12,025 articles published between 1.1.1970 and 31.8.2008. We updated the database to include additional articles published between 1.9.2008 and 31.3.2015 through combining text mining with a Markov Chain algorithm to identify a single search term. The algorithm was implemented in the statistical software environment R and executed the following steps:

1) Select all eligible English abstracts and randomly select 4000 English abstracts judged irrelevant for the review from the LERG database.
2) Remove stop words, punctuations, numbers and locations.
3) Generate word frequency vectors for both text documents (package 'tm')
4) Generate a data frame with the 100 most common words identified in the eligible abstracts.
5) Calculate relative word frequencies of the 100 terms in the eligible (p1) and selected irrelevant abstracts (p0) and calculate p1-p0.
6) Use the 7 most common words of p1 as initial key word combination (search term).
7) Calculate specificity (i.e. proportion of the selected irrelevant abstracts which contained none of the words) and sensitivity (i.e. proportion of eligible abstracts which contain at least one of the words) for the current search term.
8) Update the search term: remove or add one word of the search term. Re-calculate sensitivity and specificity. Selection probability is proportional to p1 (adding a word) or proportional to p0 (removing a word).
9) If the sum of the differences of the new and previously calculated values for sensitivities and specificities is higher than a random number drawn from the standard normal distribution, the updated search term replaces the previous one.
10) Replicate steps number seven through nine 2000 times.
11) Identify the search term with the best performance defined as sensitivity + (1-specificity).

The algorithm identified a search term with five key words which exhibited a sensitivity of (95%) and specificity of (86%) which we further combined with the leptospirosis related key words from the original search. The following search term was used in PubMed (time restriction 1.9.2008 and 31.3.2015 and species restriction 'human'):

(risk[Title/Abstract] OR workers[Title/Abstract] OR flooding[Title/Abstract] OR females[Title/Abstract] OR exposed[Title/Abstract]) AND ("Leptospira" OR "Leptospirosis" OR "Weil disease" OR "Weil's disease") NOT "Leptospirillum".

The term identified an additional 229 potentially relevant articles. Of those, 13 were considered eligible following review of the abstract or full text and have been included in the review. Two articles were identified in both searches because the electronic version was published before and the print issue was published after September 2008. The key part of the R script is provided below:

```r
###################################################
#
# Abstracts form articles included in the original review are stored in abs.1
#     and 4000 abstracts form articles excluded are stored in abs.0
# The data frame 'da' has the following variables:
#     word: list of words occurring frequently in included abstracts
#     p1: rel. frequency a certain term occurred in included abstracts
#     p0: rel. frequency a certain term occurred in excluded abstracts
#     dif: p1-p0 (data frame is sorted by dif)
#
###################################################


# functions
# drop one word from the search terms with probability proportional to p0:
exclude <- function(x){
  to.drop <- sample(x,1,prob=da$p0[x])
  return(to.drop)
}

# include one new word in search term with probability proportional to dif
include <- function(x){
  to.include <- sample(setdiff(1:100,x),1,prob=(da$dif[setdiff(1:100[1],x)]))
}

# Specificity: none of the search terms occur in excluded abstracts
sp <- function(x){
  tot.hits <- NA
  for(i in x){
    hits <- grep(da$word[i], abs.0)
    tot.hits <- sort(unique(c(hits, tot.hits)))
  }
  return(100-length(tot.hits)/length(abs.0)*100)
}

# Sensitivity: at least 1 of the search terms occurs in included abstracts
se <- function(x){
  tot.hits <- NA
  for(i in x){
    hits <- grep(da$word[i], abs.1)
    tot.hits <- sort(unique(c(hits, tot.hits)))
  }
  return(length(tot.hits)/length(abs.1)*100)
}


# initial values
rule.sd <- 1 # standard deviation for decision rule
term <- 1:7 # select the first 7 words as initial search term
se.old <- se(term) # calculate sensitivity of the initial search term
sp.old <- sp(term) # calculate specificity of the initial search term

words <- da$word[term]
length(words) <- 12 # maximum number of words is 12
res <- c(se.old, sp.old, words) # init matrix to store results (se, sp, term)
```

```r
# start the text mining loop
for(i in 1:2000){
 rand <-runif(1)
 if(rand<0.5){ # chance 50% to include 1 new word
  to.include <- include(term)
  se.new <- se(c(term,to.include))
  sp.new <- sp(c(term,to.include))
  perf <- (se.new-se.old) + (sp.new-sp.old)
  # decision rule: improvement higher than rnorm(1,0,rule.sd)->keep the change
  rule <- perf > rnorm(1,0,rule.sd)
  if(rule){
     term <- sort(c(term,to.include))
     se.old <- se.new
     sp.old <- sp.new
     words <- da$word[term]
     length(words) <- 12
     res <- rbind(res,c(se.new,sp.new,words))
  }
 }
 if(rand>=0.5){ # chance 50% to drop 1 word
  to.exclude <- exclude(term)
  se.new <- se(setdiff(term,to.exclude))
  sp.new <- sp(setdiff(term,to.exclude))
  perf <- (se.new-se.old)  + sp.new-sp.old
  rule <- perf > rnorm(1,0,rule.sd)
  if(rule){
     term <- sort(setdiff(term,to.exclude))
     se.old <- se.new
     sp.old <- sp.new
     words <- da$word[term]
     length(words) <- 12
     res <- rbind(res,c(se.new,sp.new,words))
  }
 }
}
```
