# Supplementary figures and images for: Environmental and Behavioural Determinants of Leptospirosis Transmission: A Systematic Review
Source: PLoS Negl Trop Dis. 2015 Sep 17;9(9):e0003843. doi: 10.1371/journal.pntd.0003843 (PMC4574979; doi:10.1371/journal.pntd.0003843)

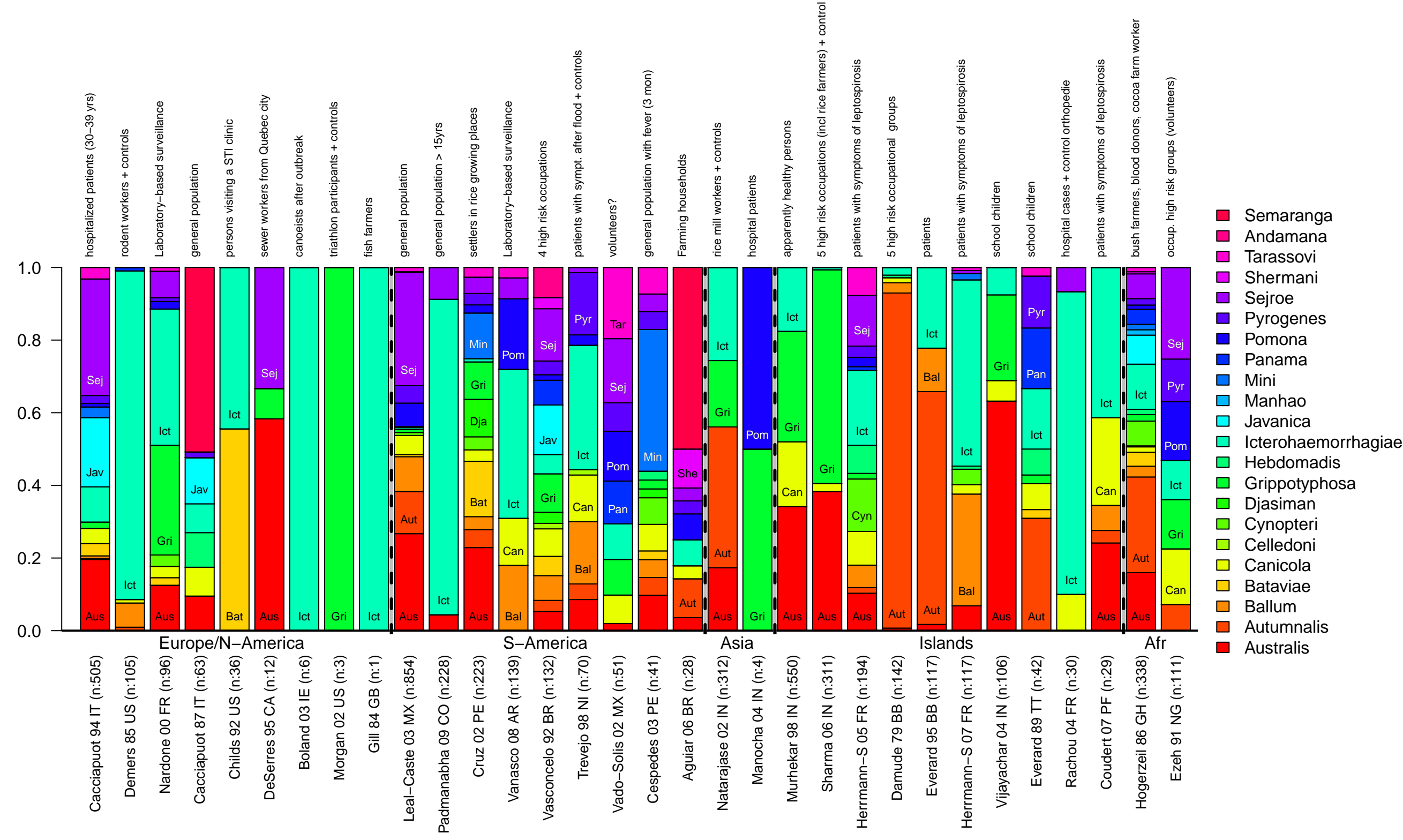

Supplement: S1 Fig — (PDF) [file pntd.0003843.s004.pdf]
