# Supplementary material for: Environmental and Behavioural Determinants of Leptospirosis Transmission: A Systematic Review
Source: PLoS Negl Trop Dis. 2015 Sep 17;9(9):e0003843. doi: 10.1371/journal.pntd.0003843 (PMC4574979; doi:10.1371/journal.pntd.0003843)
Supplement: S2 Fig — (PDF) [file pntd.0003843.s005.pdf]

PRISMA 2009 Flow Diagram

Note: A more detailed diagram is provided in the manuscript (Fig. 1)

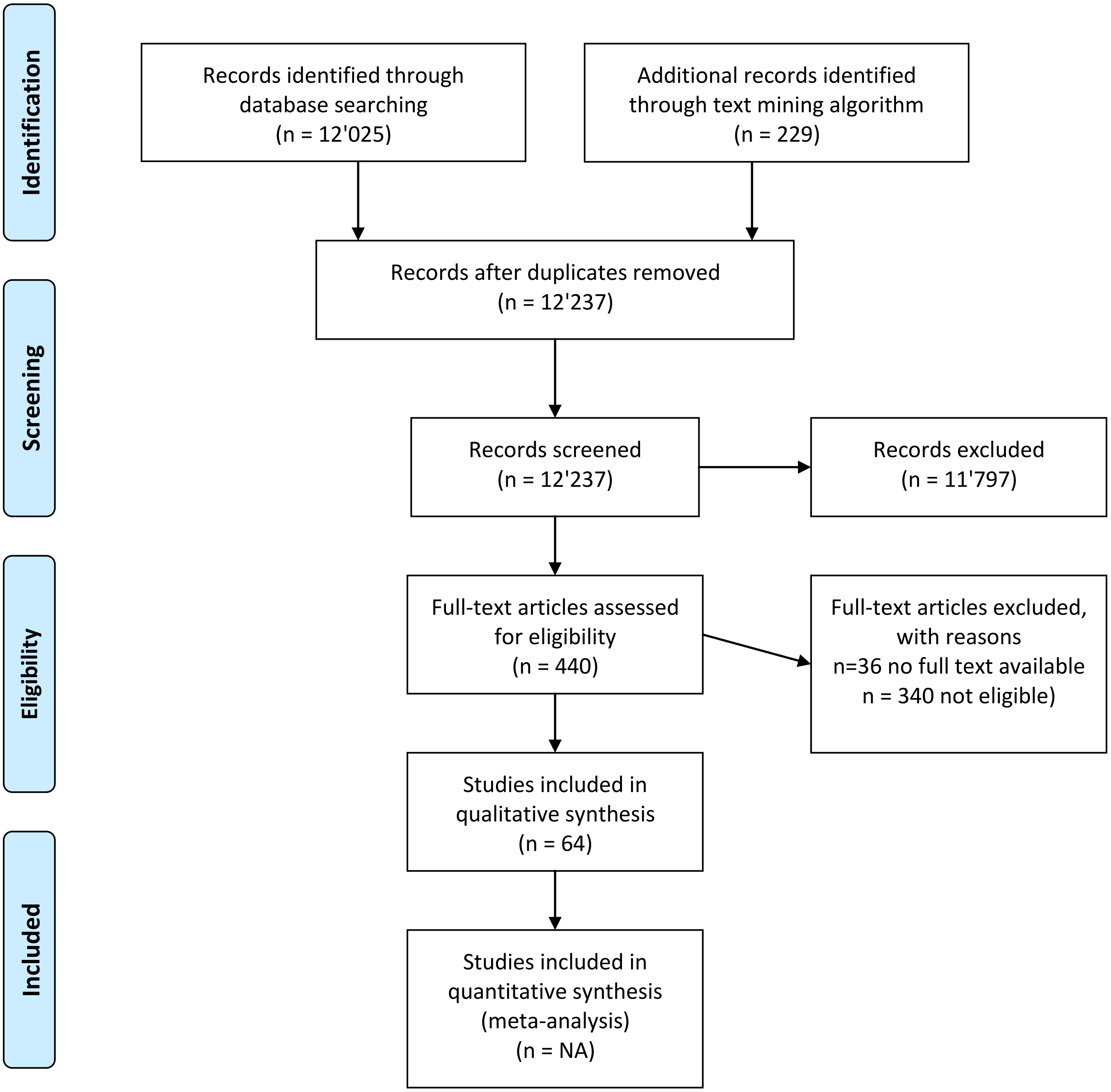

From: Moher D, Liberati A, Tetzlaff J, Altman DG, The PRISMA Group (2009). *P*referred *R*eporting *I*tems for *S*ystematic Reviews and *M*eta-*A*nalyses: The PRISMA Statement. PLoS Med 6(6): e1000097. doi:10.1371/journal.pmed1000097

For more information, visit www.prisma-statement.org.
